# DIZZYNET—a European network initiative for vertigo and balance research: visions and aims

**DOI:** 10.1007/s00415-015-7912-3

**Published:** 2016-04-15

**Authors:** Andreas Zwergal, Thomas Brandt, Mans Magnusson, Christopher Kennard

**Affiliations:** Department of Neurology, University of Munich, Munich, Germany; German Center for Vertigo and Balance Disorders, DSGZ, University of Munich, Munich, Germany; Clinical Neurosciences, University of Munich, Munich, Germany; Department of Otorhinolaryngology, Clinical Sciences, Lund University and Skåne University Hospital, Lund, Sweden; Nuffield Department of Clinical Neurosciences, University of Oxford, Oxford, UK

**Keywords:** European Dizzynet, Vertigo, Dizziness, Balance disorders, Vestibular

## Abstract

Vertigo is one of the most common complaints in medicine. Despite its high prevalence, patients with vertigo often receive either inappropriate or inadequate treatment. The most important reasons for this deplorable situation are insufficient interdisciplinary cooperation, nonexistent standards in diagnostics and therapy, the relatively rare translations of basic science findings to clinical applications, and the scarcity of prospective controlled multicenter clinical trials. To overcome these problems, the German Center for Vertigo and Balance Disorders (DSGZ) started an initiative to establish a European Network for Vertigo and Balance Research called DIZZYNET. The central aim is to create a platform for collaboration and exchange among scientists, physicians, technicians, and physiotherapists in the fields of basic and translational research, clinical management, clinical trials, rehabilitation, and epidemiology. The network will also promote public awareness and help establish educational standards in the field. The DIZZYNET has the following objectives as regards structure and content:

- to focus on multidisciplinary translational research in vertigo and balance disorders,
- to develop interdisciplinary longitudinal and transversal networks for patient care by standardizing and personalizing the management of patients,
- to increase methodological competence by implementing common standards of practice and quality management,
- to internationalize the infrastructure for prospective multicenter clinical trials,
- to increase recruitment capacity for clinical trials,
- to create a common data base for patients with vertigo and balance disorders,
- to offer and promote attractive educational and career paths in a network of cooperating institutions.

In the long term, the DIZZYNET should serve as an internationally visible network for interdisciplinary and multiprofessional research on vertigo and balance disorders. It ideally should equally attract the afflicted patients and those managing their disorders. DIZZYNET will not compete with the traditional national or international societies active in the field, but will function as an additional structure that addresses some of the above problems.

## Introduction

Over the last decades the field of vertigo and balance research has undergone fundamental changes. Its methodological armamentarium has rapidly developed and nowadays includes genetic, biochemical, imaging techniques as well as neurophysiology, mathematical modeling and robotics. Novel diagnostic tests have been introduced to clinical practice such as the video head-impulse test, the subjective visual vertical, vestibular-evoked potentials, and automated analyses of posture and gait. New pharmacological agents have been developed to treat vestibular, ocular motor and cerebellar disorders. Prospective clinical trials have become increasingly important to prove the efficacy and benefit of these agents. Quality of life and functioning aspects have been recognized as an essential part of the management of patients with vestibular disorders. The concept of higher vestibular functions has set new foci for the impact of the vestibular system on cognition, spatial orientation and memory, navigation and hemispheric lateralization.

These developments have brought new challenges. For example, the increasing demand for transfer of basic knowledge to patients has led to more studies being planned but their number greatly exceeds the current recruitment capacities of single institutions. In contrast to other medical research fields (cardiology, oncology, immunology, vascular neurology), the field of vertigo and balance has an almost nonexistent culture of prospective multicenter studies. This state of affairs can be seen in the striking rarity of such studies. It is also difficult to expand patient recruitment so as to satisfy a multicenter network because the technical equipment and study expertise for high-quality data collection are only available at a few centers. This situation is further complicated by differences in the terminology of disorders, diagnostic criteria, technical tests, and treatment concepts.

These challenges can only be overcome by an international cooperative initiative that combines collaboration and exchange among scientists, physicians and physiotherapists. Therefore, the German Center for Vertigo and Balance Disorders started an initiative to constitute a European Network for Vertigo and Balance Research under the label DIZZYNET. Thirty international experts from 13 countries participated in the founding meeting, which took place in Sonnenhausen in 2014 with the support of the German Ministry of Education and Research (BMBF).

## Previous developments and prerequisites

In 2009, the German Ministry for Education and Research (BMFB) funded the German Center for Vertigo and Balance disorders (DSGZ) within the framework of integrated research and treatment centers. The main aims of this funding initiative were to reinforce interdisciplinary cooperation, to translate basic knowledge into clinical applications, and to promote the conductance of more prospective clinical trials. Thanks to its strong scientific background and the generous funding of the BMBF, the DSGZ has since then developed into one of the leading international centers for the diagnosis and treatment of vertigo and balance disorders. The major steps taken to achieve this have included the establishment of:

- a pioneering interdisciplinary organization with flat hierarchies and a transparent decision-making process,
- a successful translational scientific environment embedding basic and clinical research,
- a longitudinal care chain within an Interdisciplinary Dizziness Clinic located at one place in the University Hospital,
- a national infrastructure for multicenter trials in vertigo and balance disorders,
- an international consensus process to define standards for functioning and quality of life in patients with vertigo and balance disorders,
- a teaching and training infrastructure that promotes careers in the DSGZ [1].

**Fig. 1:**
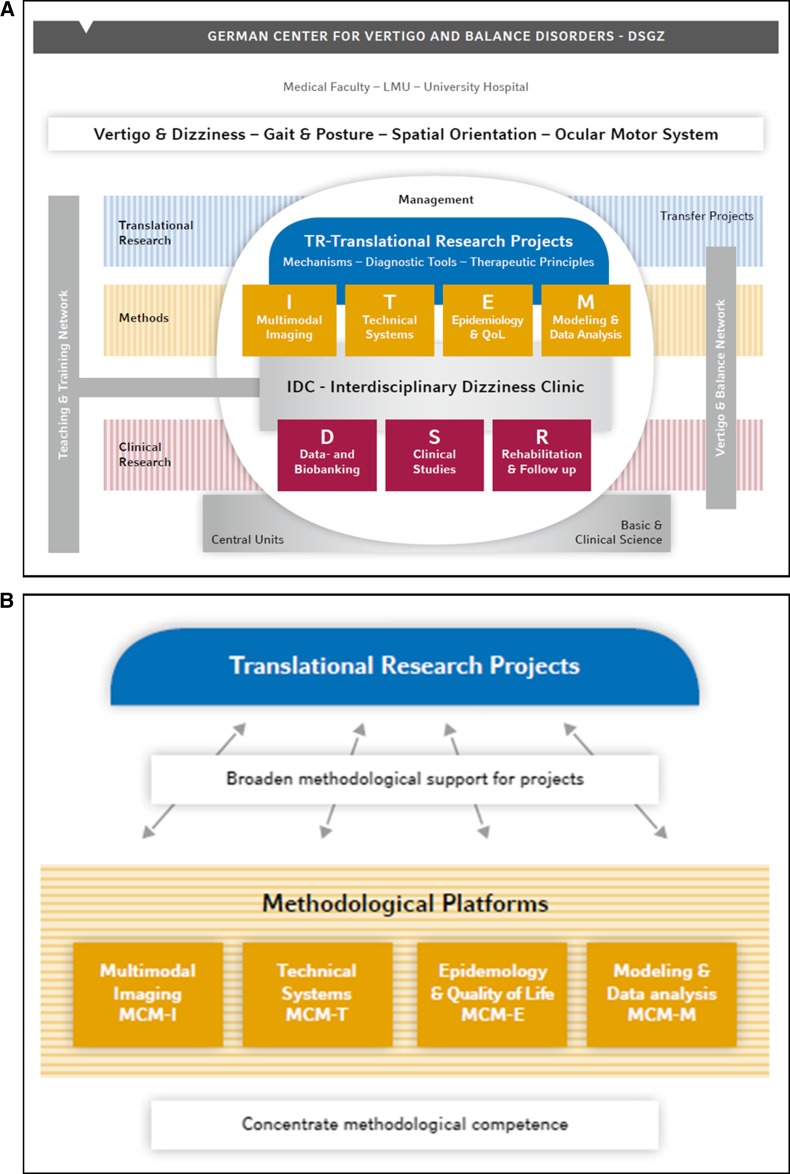
Organizational chart of the DSGZ as an independent institution of the LMU. **a** The DSGZ is an interdisciplinary clinical center under the auspices of the LMU, the Medical Faculty, and the University Hospital. Clinical research is divided into the sections diagnostics (Data- and Biobanking), therapy (Clinical Studies) and aftercare (Rehabilitation and Follow-up), which are supported by clinical research modules. Patient management takes place in the Interdisciplinary Dizziness Clinic, which offers a longitudinal care chain with gold standard diagnostic and therapeutic procedures. A Vertigo and Balance Network will organize sites all over Europe for the exchange of methods, performance of prospective clinical studies, and referral of patients. Training in the DSGZ is organized in a Teaching and Training network which offers programs for Ph.D. students, postdoc scientists, and clinicians in cooperation with existing programs. **b** Research is organized in translational research projects, which focus on four core research topics and cover aspects of pathophysiology, diagnostics, and therapy to ensure transfer of knowledge to patients. The translational research projects are supported by methodological platforms. A close interaction of translational and clinical research, patient management and teaching and training helps to overcome barriers between professions and disciplines, improve the transfer of knowledge to patients, and attract promising young scientists to the field of vertigo and balance research

Despite these achievements an intermediate analysis of strengths and weaknesses made clear that future improvement of the quality of its research, teaching, and patient care can only be accomplished by concentrating on core areas of expertise and widening the scope of scientific networking to the international level. Therefore, the following measures were proposed (Fig. 1):

- Translational research will concentrate on the four core research topics ‘vertigo and dizziness’, ‘gait and posture’, ‘spatial orientation’ and ‘ocular motor disorders’.
- Methodological platforms will strengthen the research infrastructure by bundling and advancing technical expertise, avoiding duplication and parallel development, and making methods available for all DSGZ researchers and external partners.
- Clinical studies will be performed in a multicenter context of study sites within the newly founded European DIZZYNET to guarantee optimal recruitment.
- The patient care chain of the Interdisciplinary Dizziness Clinic (IDC) will develop common diagnostic and therapeutic guidelines to optimize knowledge transfer to patients, establish concepts for tailored care, and allow structured follow-up and validation of cost-effectiveness in terms of patient-reported outcomes.
- Teaching and training will be adapted to different educational levels, skills, and needs of the career pathways of researchers and clinicians by consolidating existing scientific training programs in an international Teaching and Training Network.

In an intermediate project evaluation of the DSGZ in 2013 a board of international experts strongly recommended intensifying international networking, and the BMBF financially supported the realization of the idea of a European DIZZYNET.

## Overall concept and aims of the DIZZYNET

Vertigo and balance disorders are among the most common key symptoms in medicine. Despite this high prevalence, patients with vertigo often consult physicians of various disciplines (e.g., Neurology, Otolaryngology, Internal Medicine, Orthopedics) and undergo unnecessary diagnostic testing only to receive a false diagnosis and ineffective treatment. This situation results in substantial psychosocial harm and economic costs. The current unsatisfactory management of dizzy patients is due to the following deficits in academic medicine, medical training, and clinical research:

- a narrow view of the symptom of vertigo due to the compartmentalization of clinical specializations;
- non-uniform guidelines for diagnosis and therapy among the disciplines;
- insufficient interdisciplinary cooperation among disciplines and basic and clinical scientists;
- difficulty recruiting large-scale patient cohorts for clinical studies;
- failure to integrate experimental competence from the domains of engineering, computer scientists, psychosomatics, and quality of life research.

**Fig. 2:**
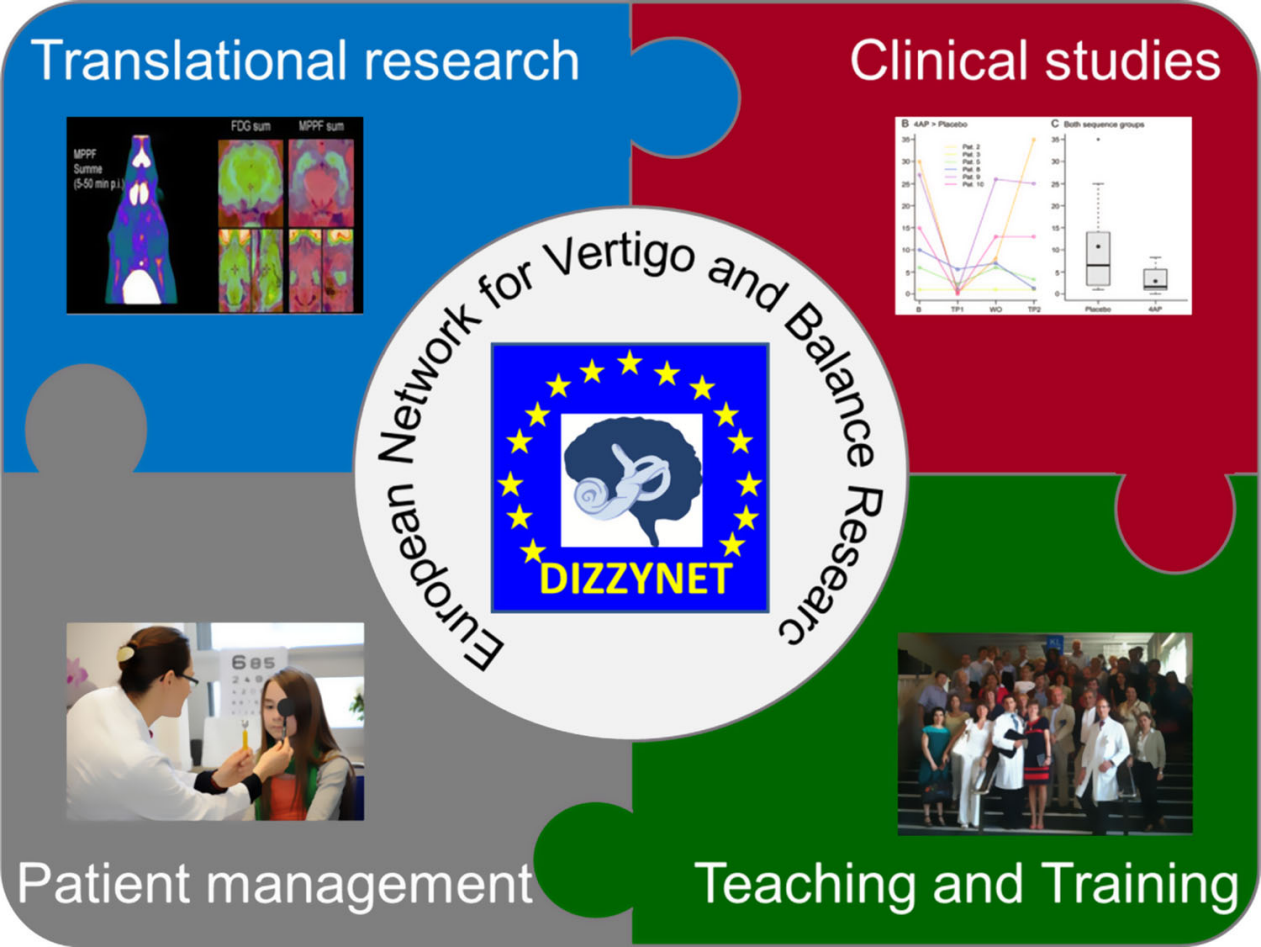
Core aspects of the DIZZYNET. A close interaction of translational and clinical research, patient management, and teaching and training will help to overcome barriers between professions and disciplines, improve transfer of knowledge to patients, and attract promising young scientists to the field of vertigo and balance research. The DIZZYNET should serve as a structural link between these fields

It is evident that these problems are also present in individual research and treatment institutions all over Europe and elsewhere. They can only be overcome by the concerted action of a network of institutions that integrate all professions working in the field of vertigo and balance research. By taking the following steps, the DIZZYNET aims to overcome these deficits and weaknesses (Fig. 2):

- create a platform for collaboration and exchange of basic researchers, clinical scientists, physicians, and physiotherapists;
- improve multidisciplinary translational research;
- cooperate with the leading societies to develop common standards in diagnostics, methods, and treatment;
- internationalize the infrastructure for prospective multicenter clinical trials;
- increase recruitment capacity for clinical trials by creating a common data base for patients with vertigo and balance disorders;
- coordinate research on rare vestibular disorders by exchanging data and specimens;
- set new foci on neglected aspects of vestibular research like rehabilitation, epidemiology, quality of life, and functioning;
- offer and promote attractive educational and career paths within a network of cooperating institutions.

**Fig. 3:**
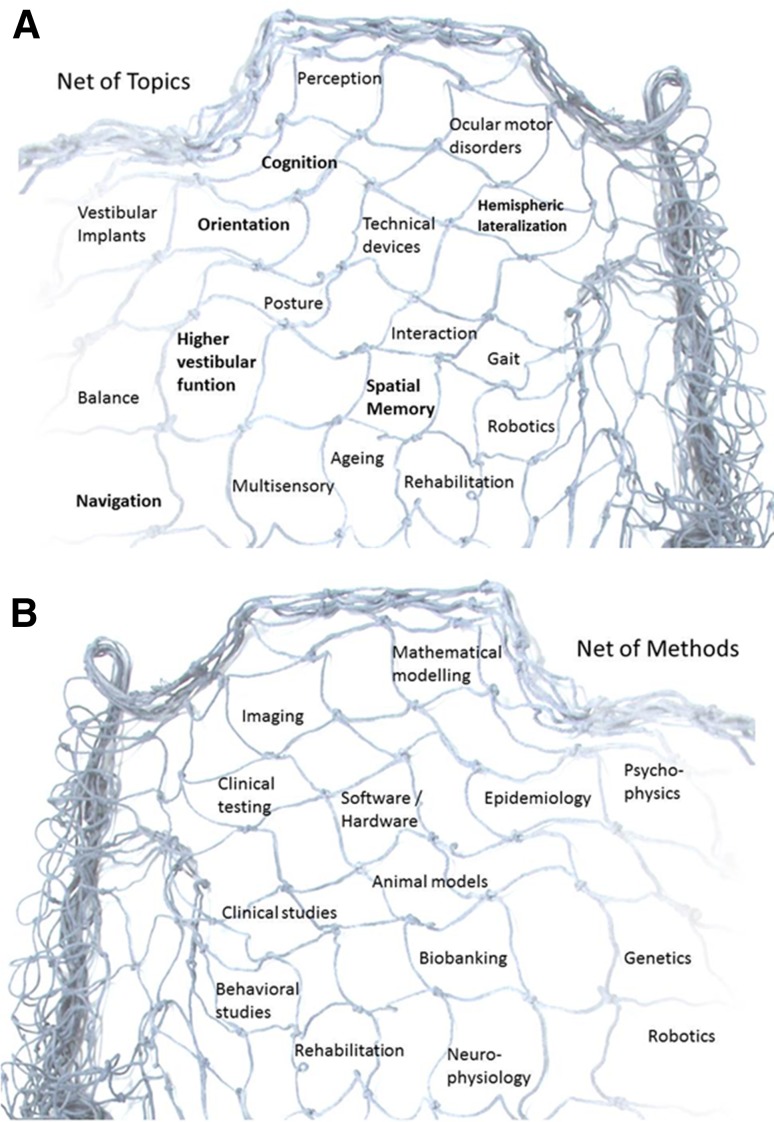
Topics and methods included in the DIZZYNET. A scientific net is not only a net of persons. It consists of many aspects and levels. **a** It is also a net of topics, whose relevance changes over time. Ocular motor disorders, for example, which have dominated clinical and vestibular research for decades, appear less attractive nowadays. New topics, for example, spatial memory, navigation, cognition, higher vestibular function, and hemispheric lateralization are rapidly attracting more interest, even eclipsing the older focus. **b** A scientific net is also a net of methods that can be shared among net members via mutual education, information, and multinational cooperation

DIZZYNET can profitably build on the previous achievements of many centers all over Europe. However, it became clear at the founding meeting of the DIZZYNET that there is a common need to join forces to set up a platform to address these outstanding problems. DIZZYNET is not only a network of people, but also a network of topics and methods (Fig. 3a). The participants at the founding meeting in 2014 formed the following working groups to identify the most pressing problems and questions, as well as to propose projects in the relevant fields:

- Basic research,
- Clinical management and research,
- Clinical trials,
- Rehabilitation,
- Epidemiology.

The thematic focus of the next meeting in 2015 will be to further implement operational structures and workflows in the network. It will address the following organizational and structural issues:

1. Definition of aims, priorities and milestones,
2. Formulation of funding initiatives,
3. Discussion and voting on bylaws, definition of membership, interaction with societies.

In the founding meeting all participants made clear that the network will be open to all interested experts. New members can be suggested and should apply by sending a CV including a letter of intent indicating their inputs to the network initiative. The exact mode of application will be defined in the bylaws. A website will be launched to ease contacting (http://www.dizzynet.eu).

It is important to emphasize that DIZZYNET is not competing with other existing traditional national and international scientific societies. A close interaction is planned, e.g., in teaching and education. Most of the founding members are also long-standing active members of several other societies like the Barany Society. As an additional organization the central goal of the DIZZYNET is to correct some of the above-mentioned structural deficits.

## Scientific networks

### Translational research

Translational research in the field of vertigo and balance disorders includes a number of new methods and professions (Fig. 3b). The most pressing challenges are the following:

- to improve the interaction between basic and clinical researchers,
- to identify translational aspects in diagnostics,
- to validate innovative therapeutic strategies,
- to back-translate clinical practice into basic research,

The DIZZYNET is dedicated to support translational research by

- bundling interdisciplinary expertise,
- acting as a liaison between investigators with different scientific backgrounds,
- helping establish and standardize innovative methods and ideas,
- exchanging methods and samples.

### Clinical trials

The growing demand for the transfer of basic knowledge to patients has led to plans for an increased number of studies. This development, however, has greatly exceeded the current recruitment capacities of single institutions, especially for acute vertigo syndromes (e.g., vestibular neuritis), which are infrequently seen during the acute phase in hospitals due to referral practice, and for rare vertigo/dizziness syndromes (e.g., episodic ataxia). Prospective multicenter studies in the field of vertigo, balance, and ocular motor disorders are almost nonexistent. Moreover, it is difficult to expand patient recruitment to cover a multicenter network because the technical equipment and study expertise for high-quality data collection are only available at a few centers. In addition, the pharmaceutical industry has only recently become interested in clinical trials in the field of vertigo, dizziness, and ocular motor disorders.

The DIZZYNET plans to take the following steps to improve the culture of clinical trials for vertigo and balance disorders:

- to increase recruitment capacity by establishing a wide network of study sites. Existing national networks will then be embedded in this European trial network. The network will be open to new participants as long as technical equipment and study personnel fulfill requirements. Thirty study sites are now planned. The study physicians will meet regularly for educational programs and courses. All partners will follow common standards when conducting large prospective studies in the field of vertigo, balance and ocular motor disorders;
- to broaden the thematic focus of its clinical study portfolio to include new types of studies (e.g., genomics, long-term follow-up) and new fields of research (e.g., gait and posture). In this way, overlaps with neighboring clinical fields will be used to establish new areas of collaboration and to gain access to well-characterized patient cohorts;
- to install a study registry for patients with rare vertigo and dizziness syndromes to facilitate study planning and initiation. In the long term this could include data from risk assessments, clinical diagnostic tests, therapeutic plans, and follow-up assessments. The need for data management of biological data sets (blood sampling, genomic data, histo-pathological data) in vertigo and dizziness patients is evident. In view of the large data sets from genomics and the specific regulatory requirements, a unique infrastructure has to be developed for such data management;
- to follow the long-term course of vertigo and balance disorders. Such longitudinal data sets should not only improve our knowledge of the prognosis of the respective diseases and their consequences for functioning, disability and quality of life, but also reveal the effects of continuous treatments. Efforts to include external study cohorts will also be intensified. All these measures should fulfill the overall aim to conduct high-quality long-term observational studies in the core fields of the DSGZ;
- to provide a study service platform;
- to support the transfer of preclinical therapeutic concepts to clinical pilot trials;
- to install a central liaison management with external partners (e.g., in industry).

## Clinical networks

Patient recruitment and referral to specialized centers currently pose problems. To improve patient flow the DIZZYNET will link high-quality centers in which patients can receive treatment near their homes. Clinicians who have been trained in the DIZZYNET centers and continue their clinical careers at other hospitals and institutions will act as satellites of this network. Regular, short hospital visits will be arranged for physicians interested in the field of vertigo and balance disorders. By this means common standards of practice in the diagnosis and treatment of patients can be readily spread all over Europe. Such visits will also lay the groundwork for the implementation of standards in clinical guidelines.

## Teaching and training networks

**Fig. 4:**
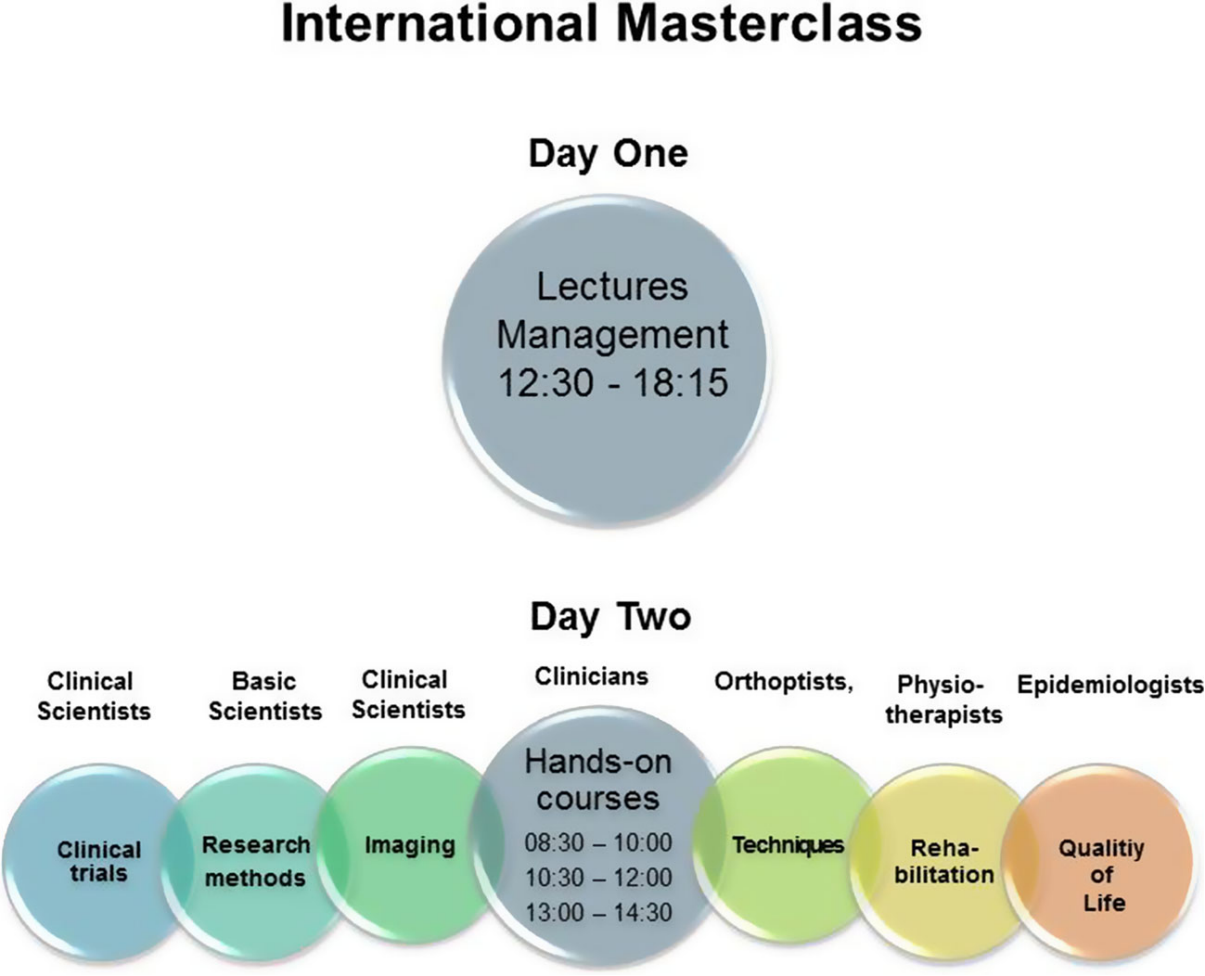
International Master Class on vestibular disorders. The yearly International Masterclass on how to manage dizzy patients and how to conduct otoneurological research has the concept: “Two Days of Teaching”. Day 1 provides lectures for all participants. Day 2 offers hands-on-courses with an occupational focus for clinicians, basic and clinic scientists, epidemiologies, orthoptists, medical technical assistants, and physiotherapists. They can select their individual menu of courses from seven blocks

Teaching and training in the field of vertigo and balance disorders aim at different target groups (researchers, physicians, technical assistants, physio- and psychotherapists). Currently only a few meetings dedicated to teaching and training take place nationally (for example, the Munich Vertigo Symposium). The DIZZYNET intends to develop more curricula tailored to the individual target groups while expanding the scope of its teaching and training program to reach all of Europe. An initial attempt is the International Master Class on vestibular and ocular motor disorders which will take place for the first time in November, 2015 and subsequently continue on an annual basis. During such meetings international experts will give lectures on the functional anatomy and clinical and laboratory examination of the vestibular and ocular motor systems as well as on how to take the history of a patient. They will also update the known clinical features and treatment of the most relevant peripheral, central, and functional vestibular as well as ocular motor disorders. Furthermore modules attractive to researchers (e.g., on research methods, imaging), technical assistants, and orthoptists (e.g., on techniques) or physiotherapists (rehabilitation of vestibular disorders) will be included (see Fig. 4).

## Achievements and long-term structural goals

The following supplement gives insight into the topics discussed at the founding meeting of the DIZZYNET in Sonnenhausen in 2014. An annual meeting of all DIZZYNET members is foreseen (second meeting in October, 2015), and working groups have been formed for the preparation. As regards teaching and training, the initiative for an annual International Master Class has already borne fruit. The first is scheduled to take place in Munich in November, 2015 (Fig. 4).

**Fig. 5:**
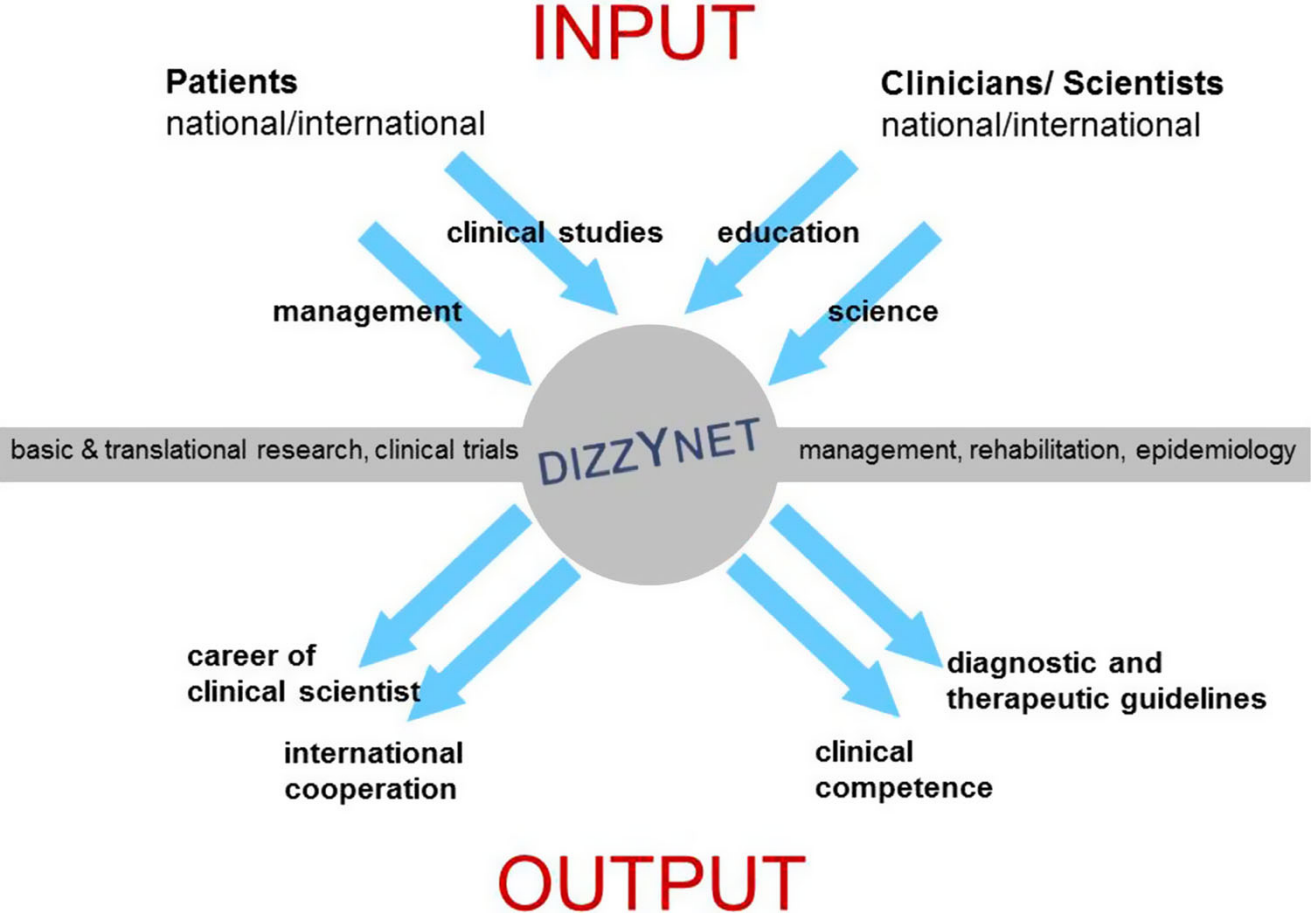
Added value of the DIZZYNET for patients and clinicians/scientists. The European DIZZYNET attracts both patients and scientists (INPUT). Patients come to receive appropriate management of their conditions and to participate in clinical diagnostic and therapeutic studies; doctors and scientists, for structured education and research on the pathophysiological mechanisms underlying the relevant disorders. The OUTPUT of the DIZZYNET on the patient’s side is clinical competence and evidence-based guidelines for diagnostic and therapeutic procedures. The OUTPUT on the scientist’s side includes the development of a topic-related career as a clinical scientist and the establishment of international, cooperative research projects

In the long term, the DIZZYNET aims to become an internationally visible platform for interdisciplinary and multiprofessional research on vertigo and balance disorders, which attracts patients, physicians, therapists, researchers, and trainees (Fig. 5). To achieve this goal it is essential to follow a concerted fundraising strategy while at the same time increasing the visibility of the network.

### Concerted fundraising strategy

In the next years, an overall fundraising strategy will be developed that identifies high-profile areas of major interest for public or private donations. The fundraising approaches will focus on the areas of research, teaching and training, and healthcare processes. For example, fundraising for research will identify clusters for future public national and international funding, establish public–private partnerships, industry-funded research initiatives, and spin-offs of practical applications in cooperation with companies, as well as attract charitable donations. Teaching and training will be funded by sponsorships of teaching events, scholarships, prizes, and applications for foundation resources that promote the careers of young scientists. Finally, fundraising to optimize healthcare processes will focus on cooperation with health insurance companies and the public health sector in the respective countries and the European Union.

### External communication and visibility

The public relations concept of the DIZZYNET will focus on branding rather than marketing and concentrate on reaching patients with vertigo and balance disorders. In the past, public response to the media presentation in the field was immense: it exceeded by far the capacities of single institutions to process the many requests. Therefore, the communication concept will now be tailored more precisely to the target groups. The overall aim will be to establish public awareness of the DIZZYNET as an international network of reference centers for patients, researchers, and industry cooperation in the field of vertigo and balance disorders. The strategy will include multiple media information for patients (radio, television, internet, http://www.dizzynet.eu) and will proactively contact highly promising researchers, potential donors, companies, and health authorities.
